# Assessment of the synthesis method of Fe_3_O_4_ nanocatalysts and its effectiveness in viscosity reduction and heavy oil upgrading

**DOI:** 10.1038/s41598-023-41441-6

**Published:** 2023-10-24

**Authors:** Parya Torkaman, Ramin Karimzadeh, Arezou Jafari

**Affiliations:** Faculty of Chemical Engineering, Tarbiat Modares University, Tehran, Iran

**Keywords:** Nanoscience and technology, Nanoscale materials, Magnetic properties and materials

## Abstract

In this research, Fe_3_O_4_ nanocatalysts were synthesized systematically microwave-assisted. The effectiveness of the synthesized nanocatalysts in reducing viscosity and upgrading heavy oil was evaluated. The nanocatalysts were investigated for their magnetic and electromagnetic properties. The impact of microwave radiation's time and power on the size and purity of nanocatalysts was investigated. The purities in the crystal network of Fe_3_O_4_ nanocatalysts expanded as a result of reducing microwave radiation time and power due to less heat production. Increased temperature leads to dope NH_4_Cl into the Fe_3_O_4_ nanocatalysts crystal network. At: 1 min and power of 400 watts the most satisfactory results in the size and purity of nanocatalysts. The electromagnetic properties, size, and effectiveness of the synthesized Fe_3_O_4_ nanocatalysts have been examined to determine the effect of the synthesis method. The performance of Fe_3_O_4_ nanocatalysts synthesized by co-precipitation and microwave-assisted viscosity reduction and heavy oil upgrading was evaluated and compared. The crystallite size of the Fe_3_O_4_ nanocatalysts synthesized by microwave-assisted was smaller than that synthesized using co-precipitation. Fe_3_O_4_ nanocatalysts synthesized by microwave-assisted and the co-precipitation method decreased viscosity by 28% and 23%, respectively. Moreover, Fe_3_O_4_ nanocatalysts synthesized by microwave-assisted reduced the sulfoxide index and aromatic index considerably more than the co-precipitation synthesized Fe_3_O_4_ (90% against. 48% and 13% vs. 7%, respectively).

## Introduction

Researchers have recently considered iron oxide nanocatalysts due to their various uses. These nanocatalysts are used in military, medical, and pharmaceutical industries, oil extraction, and water or soil treatment^1–3^. One of the reasons for the widespread use of iron oxide nanocatalysts is their magnetic properties^4,5^ Due to the properties of nanocatalysts, more attention has been paid to these materials^6–9^. The synthesis and preparation of nanocatalysts affect their properties, such as chemical composition, morphology, and particle size, and play an essential role in applying nanocatalysts in various engineering industries. Various methods are used to synthesize Fe_3_O_4_ nanocatalysts, such as sonochemical, non-hydrolytic, thermal solvent, co-precipitation, sol–gel, and microwave-assisted^10–13^.

Microwave synthesis is a fast and straightforward method performed with high yield, less reaction time, highly controlled, and energy efficiency^1,14,15^. Microwaves are an excellent way to synthesize magnetic iron oxide nanocatalysts with controlled particle size, high crystallinity, and good magnetic properties under a short reaction time^16,17^. The growth of various industries and global demand for energy resources has led to more attention to the need to use new energy resources. These include unconventional reservoirs or heavy oil reservoirs. It should be noted that using these reservoirs with conventional methods of heavy oil upgrading and recovery is challenging or unattainable^18–20^. The cost of producing, refining, and improving oil quality from these reservoirs is higher than average. In-situ upgrading of heavy oil could be an excellent way to produce and upgrade heavy oil from these reservoirs^21,22^. The basis of this method is the cracking of heavy components, reduceing the viscosity of heavy oil and increasing its mobility^23^. One new and developing method of in-site heavy oil upgrading is electromagnetic heating and microwaves. In this method, polar molecules absorb electromagnetic waves and cause molecular fluctuations and friction^24,25^. As a result of these interactions, heat is generated, and increasing the temperature will reduce the viscosity of heavy oil^18,26^. Metal oxide nanocatalysts absorbing electromagnetic waves are used to increase the penetration range of waves into the reservoirs, such as Fe_3_O_4_’, ZnO, Al_2_O_3_, NiO’, BiFeO_3_, TiO_2_, and MnO_2_^27^. In addition to absorbing electromagnetic waves and increasing the temperature by cracking heavy components such as resin and asphaltene, these nanocatalysts can likewise decline the viscosity and upgrade heavy oil^21^. Previous research has shown that iron oxide nanocatalysts have been effective among the nanocatalysts used in upgrading and oil recovery processes^28,29^.

Considering the catalytic role of Fe_3_O_4_ nanocatalysts and microwaves in upgrading heavy oil, the authors have used the simultaneous effect of these to synergize the process. It should be noted that Fe_3_O_4_ nanocatalysts synthesized by microwave-assisted have yet to be studied and investigated in upgrading heavy oil and enhancing oil recovery. In this research, Fe_3_O_4_ nanocatalysts were synthesized microwave-assisted (as stated by the effective parameters) to reduce the synthesis time and produce appropriate nanocatalysts. Then, the impact of effective parameters in synthesis by microwave-assisted on the quality and efficiency of Fe_3_O_4_ nanocatalysts was evaluated. In the initial time, the effects of the nanocatalysts synthesis by microvave-assisted have been assessed in heavy oil upgrading and viscosity reduction of heavy oil. In addition, the effect of the synthesis method on the electromagnetic properties, size, and effectiveness of the synthesized Fe_3_O_4_ nanocatalyst has been examined. The performance of the Fe_3_O_4_ nanocatalyst synthesized by co-precipitation and microwave-assisted in viscosity reduction and heavy oil recovery was evaluated and compared.

## Material and methods

The present study investigated the Fe_3_O_4_ nanocatalysts synthesis microwave-assisted and, presented the effect of this synthesized nanocatalyst on the heavy oil upgrading by the microwave heating process. Iron (III) hexahydrate (FeCl_3_.6H_2_O), iron (II) chloride tetrahydrate (FeCl_2_.4H_2_O), and ammonia solution (NH_4_OH) were prepared from Merck and Loba Chemie. All materials had an analytical grade and were used directly in this work without further purification and modification. Deionized distilled water (DI) and high-purity nitrogen (N_2_) were used. The sample of heavy crude oil from one of the oil reservoirs in southern Iran (19.95 API, density 0.9343 gr/cm^3^and viscosity 295 mPa.s @ 25 °C and 11.7% wt asphaltene content) was used for performing EM absorption. So far, no systematic study has been done on the Fe_3_O_4_ nanocatalysts synthesis by microwave-assisted and its effect on the microwave heating process; at first, the effective parameters in the synthesis using microwave were determined. Two parameters of radiation time and microwave radiation power were considered the essential effective parameters in this synthesis method. With the help of the general factorial method, experimental design for synthesis was performed by the design expert software.

### Effectual parameters and experiment design

Previous results showed that microwave power and heating time were the main parameters controlling nanocatalyst size and magnetic properties^10^. The general factorial method was used to investigate the effect of critical parameters on microwave-assisted synthesis and find the optimal combination of synthesis parameters. In this study, two quantitative parameters, power (watts) and microwave radiation time (minutes), were considered to design the experiment using the DOE software. The radiation power parameters (Parameter 1) were assessed at two levels (400 and 800 watts) and the irradiation time(Parameter 2) at three levels (1 min, 2.5 min, and 5 min), presented in Table 1. Design expert software was used to design the tests. DOE design responses (General Factorial Design) were considered for each experiment's purity of Fe_3_O_4_ and particle size distribution.

**Table 1:** Effectual parameters and their levels.

	Level 1	Level2	Level3
Parameter 1			
Power (watts)	400	800	
Parameter 2			
Time (min.)	1	2.5	5

### Synthesis of Fe_3_O_4_ nanocatalysts

Initially, 3.43 g of FeCl_2_.4H_2_O and 9.34 g of FeCl_3_.6H_2_O were added to the beaker, and 160 ml of deionized water. Also, based on previous research, preheated deionized water was used to synthesize Fe_3_O_4_ nanocatalysts^10^. The synthesis steps were continued as follows. After adding the precursors to the preheated deionized water, (at 50 °C), the mixture was placed in a microwave oven and stirred with a mechanical stirrer. After arranging the time and power of the microwave, as maintained by Table 1, the solution is irradiated with a microwave. When the synthesis is done, the solution is gradually mixed with 25 ml of 30% ammonia solution until the pH of the solution reaches 11. The microwave irradiation time was applied in a pulse, and the microwave was turned on for 30 s and then turned off for 30 s until the selected time expired. This cycle continued until the pH of the solution was 11. At this stage, a black deposit is formed. After cooling, the solution was taken out from the microwave oven and washed several times with deionized water using magnetic decantation (a permanent external magnet, 0.5T) until the pH of the solution was 7, then dried in an oven at 70 °C for 12 h. In addition, Fe_3_O_4_ nanocatalysts have been synthesized by the co-precipitation approach^29^. Furthermore, the implementation of the nanocatalysts synthesized by both methods has been measured. As well as several analyses were performed to characterize the synthesized nanocatalysts.

### Experimental setup

In this study, the modified home microwave (Feller MW-305) was employed for the radiation of electromagnetic waves at the frequency of 2.45 GHz. An infrared thermometer (MASTECH MS6531C) that could measure temperature remotely was utilized. The effect of the Fe_3_O_4_ nanocatalysts in the microwave heating process was investigated. 50 ml of the oil sample was exposed to microwave radiation at 400 watts. Furthermore, 0.1% by weight of synthesized Fe_3_O_4_ nanocatalysts was dispersed in 50 ml of a crude oil sample. Then the samples were exposed to microwave radiation at 400 watts in a time interval of 0 to 4 min. Finally, the samples were cooled to reach the ambient temperature, and their viscosity was taken at the ambient temperature.

## Result and discussion

### Characterization

#### XRD analysis

**Figure 1:**
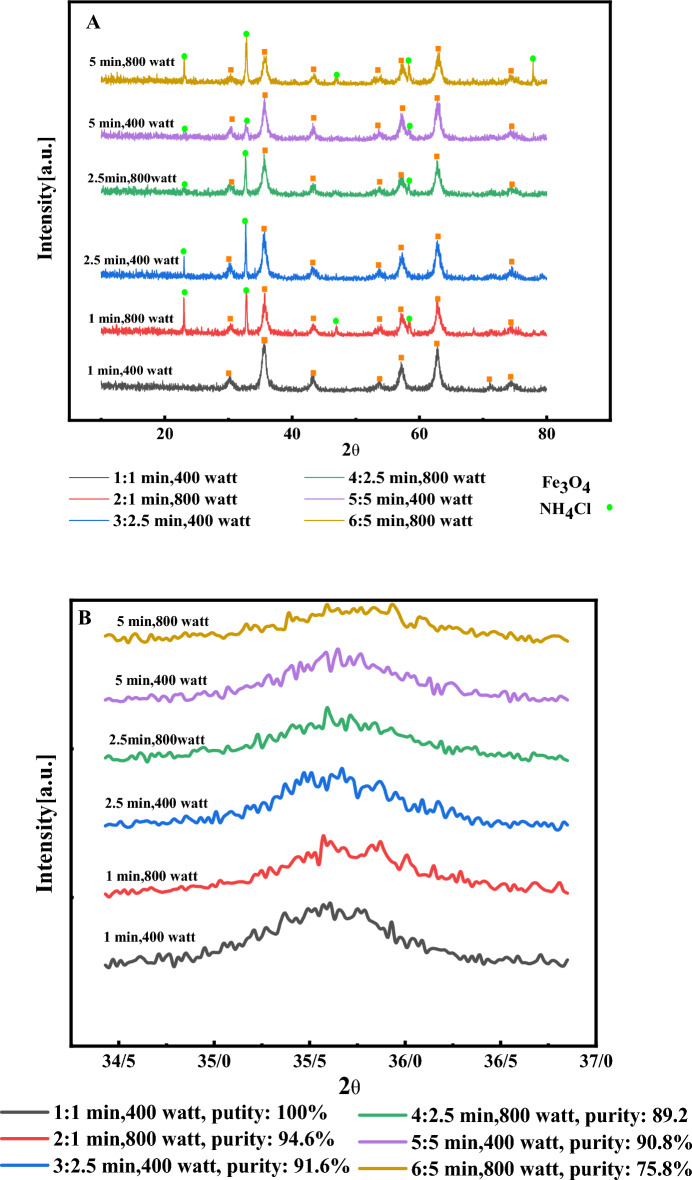
(**A**) XRD patterns of synthesized Fe_3_O_4_ nanocatalysts samples, (**B**) Enlargement of the diffraction intensity from a plane (311) in the samples.

The type of nanocatalysts, texture, crystal orientation, and crystallite size were determined using XRD (X-Ray Diffraction) analysis. Figure 1 shows the XRD pattern of Fe_3_O_4_ nanocatalysts. By use of the JCPDS No. 00-003-0862, the diffraction at 2θ = 30.17°, 35.5°, 43.3°, 53.6°, 57.6°, and 62.3° corresponds to (220), (311), (400), (422), (511) and (440) crystalline plates of Fe_3_O_4_ nanocatalysts^30–32^ respectively. Consequently, the production of Fe_3_O_4_ nanocatalysts could be concluded (Fig. 1A). During synthesizing of Fe_3_O_4_ nanocatalysts, NH_4_Cl is formed as a by-product and released by washing the nanocatalysts. However, in some conditions of syntheses, NH_4_Cl was not removed by washing. The purity is specified in each of the samples (Table 2). It should be noted, the XRD analysis was performed to determine the crystallite size distribution. The average crystallite size of Fe_3_O_4_ nanocatalysts synthesized using the Scherer equation (Eqs. 1) in different synthesis conditions is estimated between 8 and 12 nm. The magnetic behaviour of Fe_3_O_4_ nanocatalysts is sensitive to the shape and crystallinity size. When the size of nanoparticles is sufficiently small nanoparticles become superparamagnetic and they could have better saturation magnetism and improve the efficiency of nanocatalysts. However, other parameters such as synthesis temperature, synthesis method, type of precipitating material, etc. will also have an effect on the magnetic properties of these nanoparticles. Nguyen MD et al.^1^ investigated the effect of size on superparamagnetization of nanoparticles and saturation magnetism and presented similar results.

**Table 2:** Conditions of synthesis, purity, and average crystallite size in each of the samples.

No	Power of radiation (watts)	Time of radiation (min)	Final temperature of synthesis (°C)	Purity of Fe_3_O_4_ nanocatalysts (%)	Crystallite Size of Fe_3_O_4_ nanocatalysts (%)
1	400	1	52	100	8
2	800	1	56	91.3	10.6
3	400	2.5	53	91.6	12.3
4	800	2.5	61	89.2	8.5
5	400	5	58	90.8	11
6	800	5	70	75.8	9.9

As claimed by Fig. 1B, with increased power and time in microwave-assisted synthesis, a peak shift towards larger angles appeared in nanocatalysts, which increased the distance between the plates (311) and the lattice constant. During the synthesis by microwave-assisted, with the growth in power and radiation time, the temperature rises, which induces doping NH_4_Cl in the Fe_3_O_4_'s nanocatalysts^33^. It is primarily due to the extensive ionic radius of NH_4_^+^ (143 pm) compared to Fe^+3^ (64.5 pm) which is predicted to extend the crystal^34,35^. Furthermore, doping has influenced and decreased the intensity of diffraction peaks to peak displacement. It indicates that doping could decline the crystal order of Fe_3_O_4_ nanocatalysts. This tendency of modifications is compatible with the results of the XRD analysis.

As mentioned, with the increased time and radiation power in the performed syntheses, the presence of NH_4_CL increased in the structure of Fe_3_O_4_ nanocatalysts. It should be noted that in the process of electromagnetic or microwave heating, with rising radiation power and irradiation time, the sample temperature, the amount of heat produced, and the depth of penetration of waves into materials, especially in electromagnetic absorbers, will increase^36,37^. According to the results presented in Table 1, this increase in temperature during synthesis is confirmed by raising the power and irradiation time. Due to the proposed results, it could be concluded that with increasing the time and power of microwave radiation, the synthesis temperature rises, which causes an increase in impurities in the samples and dopping NH_4_Cl to the Fe_3_O_4_ nanocatalysts.

In accordance with Fig. 1B and Table 2, it could be found that increasing the time and power of microwave radiation during the synthesis of impurities NH_4_Cl is seen ascending in the five samples (No 2, 3, 4, 5, and 6). It should be noted that all samples have been washed frequently using sufficient deionized water to conclude that NH_4_Cl is not eliminated by washing, and it dops on the Fe_3_O_4_ nanocatalysts. The prominent peaks' intensity and displacement would be examined to investigate and prove this issue^33,34^ The most substantial diffraction peak from the plane (311) to investigate the peak shift by altering the purity of the samples was determined to examine the structural changes of the crystal unit. Figure 3 shows the magnification of the diffraction intensity from the plane (311). According to Fig. 1B, the top of the peak changes with increasing impurity towards a greater angle. It means that the network space between the planes (311) changes, and the network space diminishes with further development of impurities^38^.

#### FESEM analyses

**Figure 2:**
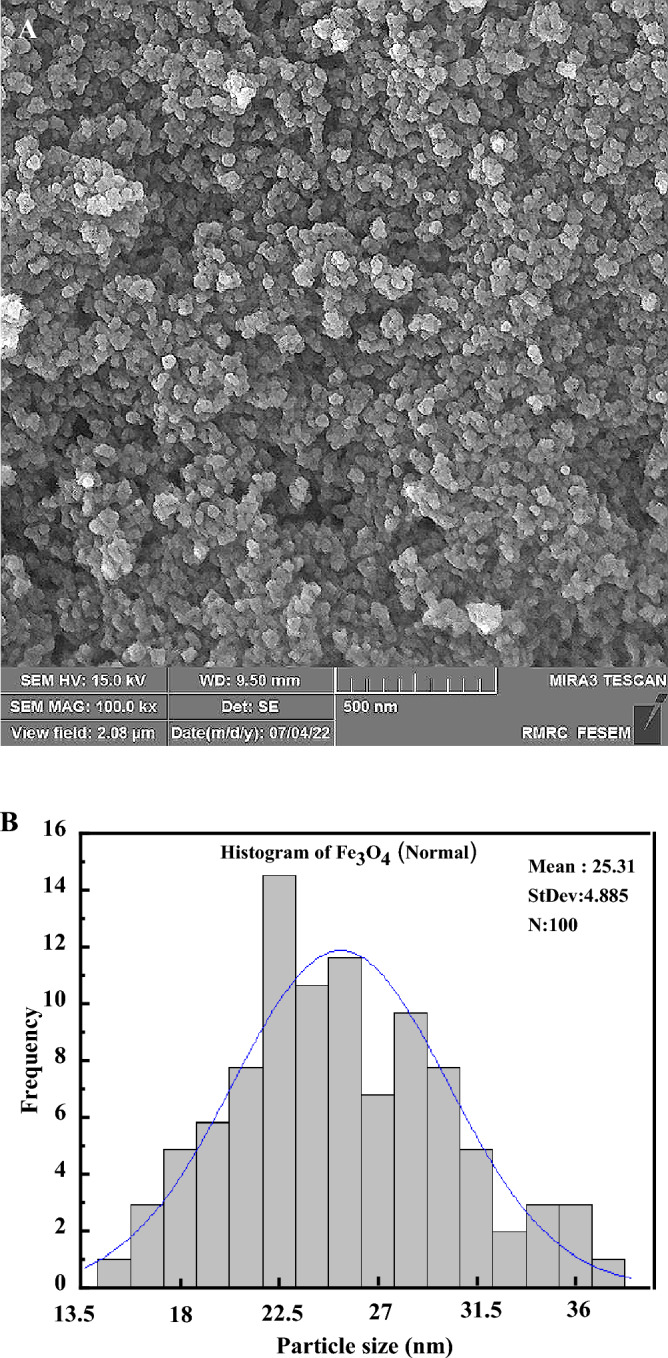
(**A**) FESEM image of Fe_3_O_4_ nanocatalyst synthesized microwave-assisted, (**B**) The average size of Fe_3_O_4_ nanocatalyst synthesized microwave-assisted.

Due to the results obtained from the XRD results and the purity of the samples, the synthesized sample was further investigated at a radiation time of 1 min and power of 400 watts using FESEM (Field Emission Scanning Electron Microscope) analysis. Figure 2A shows the representative FESEM image of Fe_3_O_4_ nanocatalysts synthesized microwave-assisted at a power of 400 watts and an irradiation time of 1 min, which had the most promising result in terms of purity. The spherical structure of synthesized Fe_3_O_4_ nanocatalysts is observable in the figure. It emphasizes the high efficiency of the synthesis method in this work for producing Fe_3_O_4_ nanocatalysts. The average size of synthesized Fe_3_O_4_ nanocatalysts can be 25 nm (Fig. 2B).

#### Electromagnetic properties

**Figure 3:**
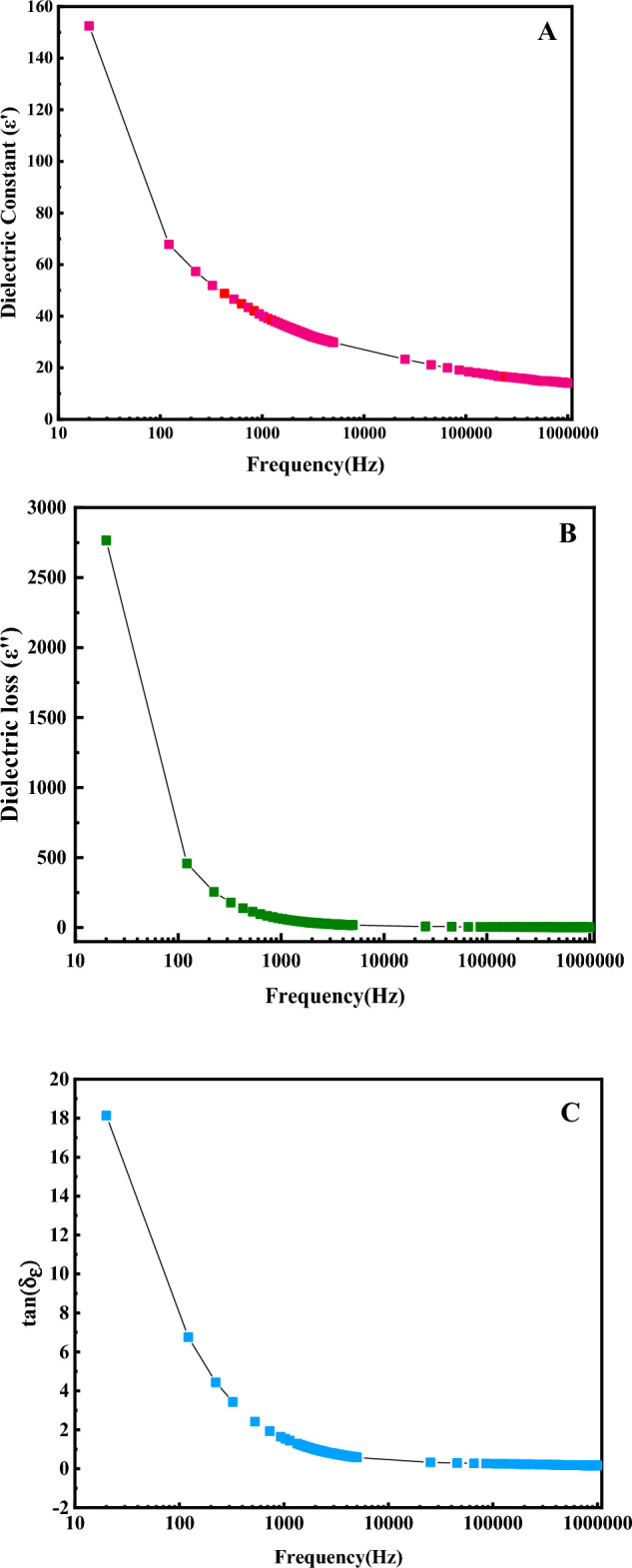
Changes of the electromagnetic properties of Fe_3_O_4_ nanocatalysts synthesized by microwave-assisted at various frequencies: (**A**) dielectric constant (ε′), (**B**) dielectric loss (ε″), (**C**) Loss tangent (tanδ_ε_).

The Electromagnetic properties of the synthesized sample (at a radiation time of 1 min and power of 400 watts) were examined. The expression ε' describes the dielectric constant and displays the ability of a compound to polarize due to an external electric field. The term ε'' is the dielectric loss, the efficiency of transforming EM energy into warmness. The electrical conductivity changes of Fe_3_O_4_ nanocatalysts versus frequency are illustrated in Fig. 3A. As maintained by the figure and the slope of the dielectric constant actual part curve (ε'). The nanocatalysts are satisfactorily affected by the electromagnetic field and are well polarized under these conditions, and similar results were reported by Gharibshahi et al.^21^.

The imaginary term electrical permittivity (dielectric loss: ε'') indicates the ability of materials to absorb electromagnetic waves. According to Fig. 3B, the actual part of the electrical permittivity (ε’) demonstrates the polarization of the nanocatalysts under the electromagnetic field. The imaginary part has a significant slope. Therefore, iron oxide nanocatalysts have acceptable efficiency in absorbing electromagnetic waves.

The loss tangent (tanδ) determines the ability of a material to convert electromagnetic energy into heat at a specific frequency and temperature. Figure 3C indicates the changes in the loss tangent in a frequency range. The more the tangential substantial losses, the more time it could absorb more energy during the electromagnetic heating process and transmit more heat and energy to its surroundings. One of the effective parameters in increasing the capacity of nanocatalysts to absorb and scatter heat in the electromagnetic process is their particle size distribution. Due to the results presented in Fig. 3C and the smaller crystallite size of Fe_3_O_4_ nanocatalysts synthesized microwave-assisted compared to Fe_3_O_4_ nanocatalysts synthesized by the co-precipitation method used by Gharibshahi et al.^29^. Therefore, Fe_3_O_4_ nanocatalysts synthesis microwave-assisted has more ability to absorb and dissipate heat in the electromagnetic heating process. These nanocatalyst has a hopeful potential for use in the electromagnetic heating process and could assist in dissipating heat in heavy oil reservoirs, cracking heavy component, reducing viscosity, and enhancing increasing heavy oil recovery.

#### Magnetic property measurements

**Figure 4:**
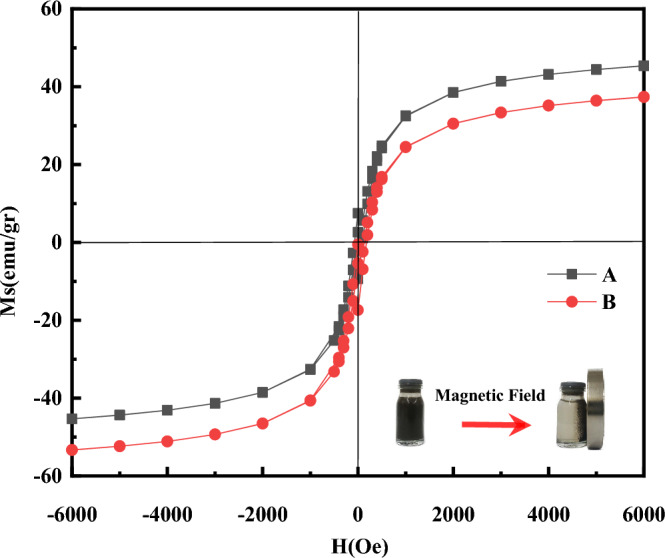
Result of VSM analysis and magnetic hysteresis loops, (**A**) Fe_3_O_4_ nanocatalysts synthesis by microwave assisted, (**B**) Fe_3_O_4_ nanocatalysts synthesis by co-percepitation.

To examine the magnetic properties of Fe_3_O_4_ nanocatalysts (synthesized by microwave-assisted at 400 watts and 1-min irradiation and synthesized by co-perception), the magnetic curves of Fe_3_O_4_ nanocatalysts were analysed by VSM analysis at room temperature (300 K) by applying field − 6000 to 6000 Oe (Fig. 4). The synthesized samples were found to have a strong magnetic response to a variable magnetic field. In Fig. 6, according to the VSM results of Figure S-type of Fe_3_O_4_ nanocatalysts, it could be seen that all of these nanostructures are supra-magnetic, and the M-H curves of the Fe_3_O_4_ nanocatalysts samples show a nonlinear characteristic with residue (Fig. 4). The saturation magnet of the Fe_3_O_4_ nanocatalysts synthesized by microwave-assisted had 46.33 emu/g and Fe_3_O_4_ nanocatalysts synthesized by co- precipitation had 37.36 emu/g. The quantity of saturation magnetization of the Fe_3_O_4_ nanocatalysts is 34.5 emu/g^39^. Due to Fig. 4, the saturation magnetization, of Fe_3_O_4_ nanocatalysts synthesized by microwave is more than nanocatalysts synthesized using the co-precipitation method. One of the important parameters in determining saturation magnetization is the crystallite size, and the smaller the crystallite size of the nanoparticles, the better they will be affected by the magnetic field and the more saturation magnetization they will have. Therefore, the smaller crystalline of the nanocatalyst synthesized by microwave compared to the co-precipitation method (8 nm vs. 18 nm) has caused more saturation magnetization of these nanoparticles. In other words, microwave synthesis could improve the magnetic properties due to the reduction of crystallite size.

### Data analysis

**Figure 5:**
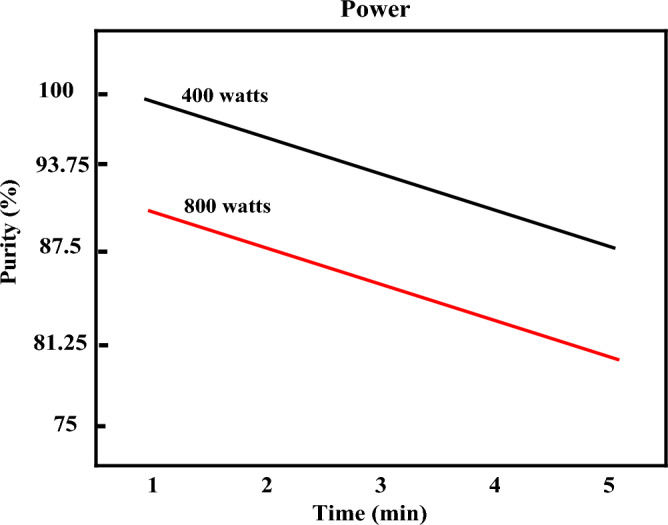
Interaction effect of power and time of irradiation on purity.

As stated, the synthesis of Fe_3_O_4_ nanocatalysts was carried out according to the general factorial design. The results of the ANOVA Table are presented in Table 2-S. Figure 5 shows the interaction between the power and time of radiation during the synthesis of the purity of Fe_3_O_4_ nanocatalysts. Based on this figure and the parallelism of the two graphs, it could be concluded that the interaction between the power and the radiation time is low. Also, with increasing power and time, the amount of purity has decreased. It is because with the growth in power and time, the amount of heat produced during the synthesis boosts, and the opportunity for nucleation and growth of Fe_3_O_4_ nanocatalysts decreases. As a result, the final purity will decrease. Finally, the optimal point was determined by the conditions of radiation power: 400 watts, radiation time: 1 min, purity of Fe_3_O_4_ nanocatalysts: 99.74, and Desirability: 0.99. Moreover, the optimal point validation test was performed, and its results are presented in Table 1-S.

### Examination of the influence of irradiation time and power in microwave-assisted synthesis

**Figure 6:**
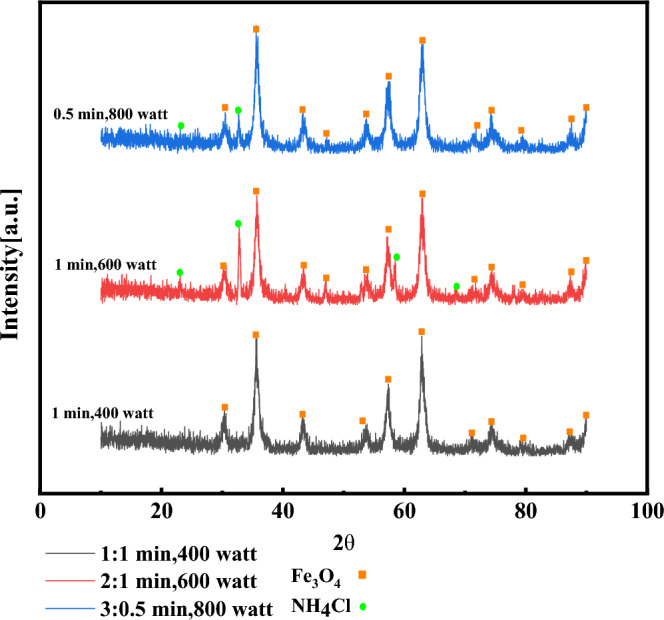
Investigation of power and time effect by analyzing XRD patterns of synthesized Fe_3_O_4_ nanocatalysts samples.

Since in the first stage of the synthesis, the synthesis by power conditions of 400 watts and the irradiation time of 1 min had a satisfactory result and was free of impurities, the repeatability of this synthesis was investigated, and its results are presented in Table 1-S. Moreover, considering that by raising the irradiation time of microwave waves during synthesis for more than 1 min and 400 watts, to ensure the effect of power and irradiation time in microwave-assisted synthesis, two syntheses were performed with the conditions presented in Table 2-S. The results of the relevant analysis are presented in Fig. 6. By improving the time and power of microwave radiation, the amount of heat produced during the synthesis process increases, and this issue causes unwanted compounds (NH_4_Cl) to dope on the Fe_3_O_4_ nanocatalysts crystallite network. As stated by the results presented in Fig. 6 and the comparison of the synthesis conditions, it can be seen that the effect of radiation power on microwave-assisted synthesis is more critical than the time of microwave radiation. By increasing the power from 400 to 800 watts, even though the irradiation time is reduced to 30 s, NH_4_Cl has doped on the Fe_3_O_4_ nanocatalysts. At a fixed time of one minute, with the increase of power from 400 to 600 watts, the presence of NH_4_Cl can be seen again. Due to the peak intensity at 2θ = 32.65, Associated with NH_4_Cl, It could conclude that the effect of growing the power in creating NH_4_Cl, and its doped Fe_3_O_4_ nanocatalysts would be more significant than improving radiation time.

## Effect of electromagnetic radiation on temperature and viscosity deviation

**Figure 7:**
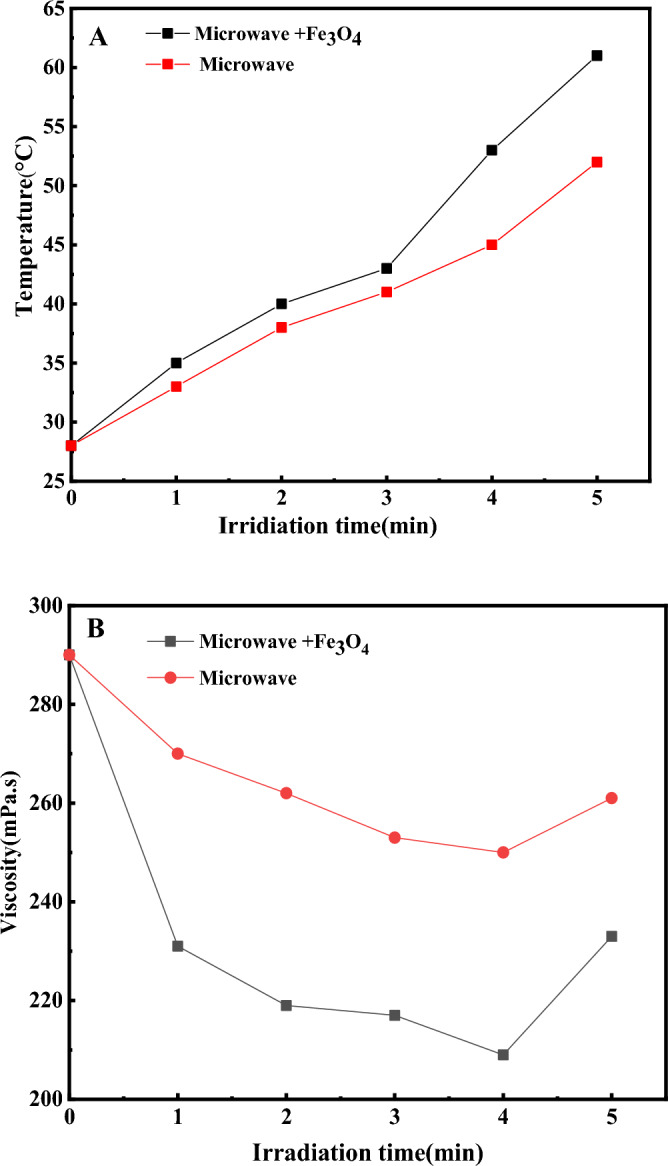
(**A**) Temperatutr variation under MW radiation (400 watts) with 0.1% wt and without Fe_3_O_4_ nanocatalysts, (**B**) Viscosity variation under MW radiation (400 watts) with 0.1% wt and without Fe_3_O_4_ nanocatalysts.

Figure 7A presented temperature variation under MW radiation (400 watts) with 0.1% wt and without Fe_3_O_4_ nanocatalysts. The electromagnetic field causes movement in free and bonded charges (electrons and ions) and dipoles, resulting in resistance to induced motion, which could lead to energy loss due to friction, elastic and inertial forces, and heating of materials. During microwave heating, the material's absorption of electromagnetic energy will enhance the sample's temperature. The capability to absorb electromagnetic waves is an essential parameter in raising the efficiency of the electromagnetic heating process^18^.

According to Fig. 7A, with the increase in the radiation time of the waves, the temperature of both samples increased because the amount of heat produced during the process raised, which led to an increase in the temperature. Also, increasing the temperature causes the cracking of heavy components and a reduction in the viscosity of the samples. That, in the Fig. 7B, this issue is quite clear. On the other hand, the oil sample containing Fe_3_O_4_ nanocatalysts has a more significant decrease in viscosity than the sample without nanocatalysts. Since Fe_3_O_4_ nanocatalysts are electromagnetic wave absorbers with a high ability to absorb electromagnetic waves and convert them into heat, as well as, the viscosity decreases as the temperature increases.

Furthermore, another experiment was designed and performed to compare the effect of the synthesis method (microwave-assisted and co-precipitation method) on nanocatalyst efficiency in the electromagnetic heating process with microwave waves. It should be mentioned that Fe_3_O_4_ nanocatalysts were synthesized by the co-precipitation method according to the method presented in Ref.^29^. As stated in Fig. 7B, the highest viscosity reduction was reported in 4 min and then the viscosity was raised. Therefore, this time could be the optimum point to check the effect of the type of synthesis method. In 4 min and 0.1% wt of nanocatalysts, the effect of the nanocatalyst synthesis method and the presence of waves were investigated.

**Figure 8:**
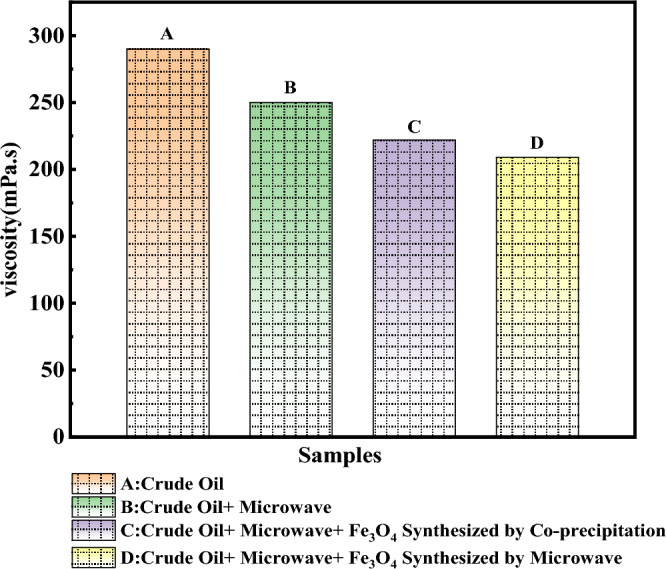
Investigation of synthesis method in Oil viscosity variation under MW radiation at power400 watts and adding 0.1 wt% of synthesized Fe_3_O_4_ nanocatalysts for all oil samples.

It should be noted that after 4 min of microwave irradiation, the temperature of the crude oil sample, the temperature of the crude oil sample with the Fe_3_O_4_ nanocatalyst synthesized by the co-precipitation method, and the temperature of the crude oil sample with the Fe_3_O_4_ nanocatalyst synthesized by microwave-assisted were 45, 50 and 53 °C respectively. That, the amount of weight, loss of the samples due to the increase in temperature was very negligible. The results have been presented in Fig. 8. According to Fig. 8, the crude oil sample, the crude oil sample containing the nanocatalyst synthesized by the co-precipitation method, and the crude oil sample containing the nanocatalyst synthesized using microwave-assisted caused a decrease in viscosity by 11, 23, and, 28%, respectively. The nanocatalyst synthesized with the microwave-assisted has caused a more considerable viscosity reduction than the nanocatalyst synthesized by the co-precipitation method, and it is more effective in reducing viscosity and upgrading heavy oil with microwave heating.

This difference could be because the nanocatalysts synthesized by the microwave-assisted have a smaller crystallite size and more prominent absorbing microwaves than the nanocatalysts synthesized by the co-precipitation method. The crystallite size of the nanocatalyst microwave-assisted synthesized in this research was 8 nm (Table 2). The crystallite size of the nanocatalyst synthesized by the co-precipitation method was 18 nm. It is the cause of the higher efficiency of these particles in the microwave heating process, increasing the temperature and reducing viscosity.

**Figure 9:**
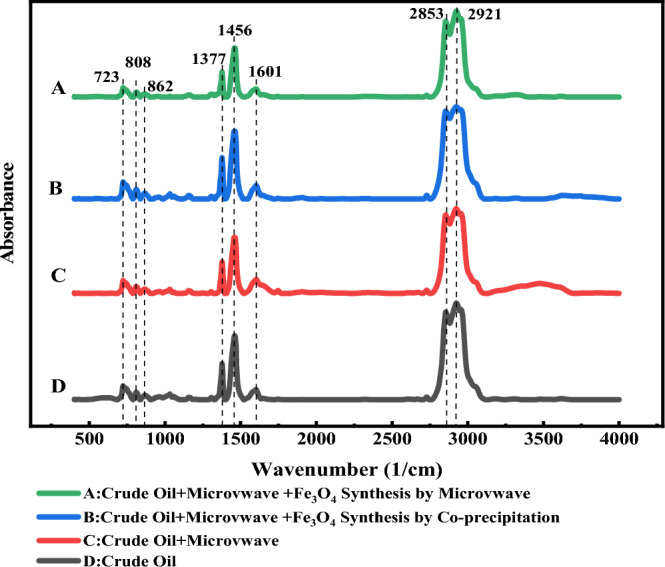
FTIR spectra of all the crude oil samples.

FTIR analysis was used to investigate the upgrading of heavy oil and changes in its composition. As stated previously, according to Fig. 7-B, the maximum decrease in the viscosity of crude oil samples was reported in 4 min. Therefore, in 4 min of irradiation of a microwave, power 400 watts and 0.1% by weight of nanocatalyst Fe3O4 nanocatalysts, the effect of the nanocatalyst synthesis method and the presence of waves on the improvement and the composition of the oil were investigated. The results are presented in Fig. 9. With the help of the results of FTIR, the Functional group indexes were investigated in different samples. In this study, these indexes are aromatic, aliphatic, branched alkane, and sulfoxide. As stated in Fig. 9, the mentioned indices were calculated in the crude oil sample, the crude oil sample under wave irradiation, the crude oil sample containing nanocatalyst synthesized by the co-precipitation method under wave irradiation, and the crude oil sample containing nanocatalyst synthesized using microwave-assisted under wave irradiation. Functional group indexes are calculated from the band area instead of band height because the peak towers are very close to each other. Band area ratio provides the possibility to calculate and compare several types of indicators. These indices were used to determine and compare the chemical composition of each component.

**Figure 10:**
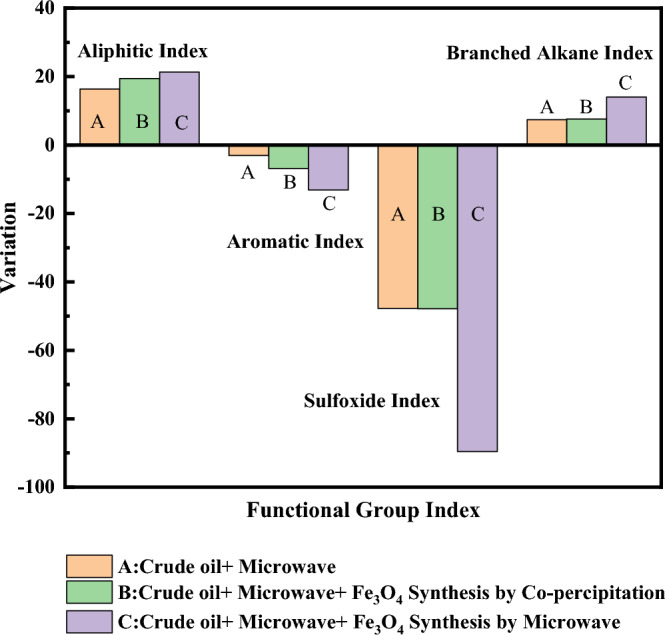
Variation of functional group under MW radiation (400 watts) with 0.1% wt and without Fe_3_O_4_ nanocatalysts.

The indicators that have been examined in this research are as follows. Aromatic index (${(A}_{1600})/(\sum A)$), Aliphatic index (${(A}_{1376}+{A}_{1460})/(\sum A)$), Sulfoxide index (${(A}_{1030})/(\sum A)$) and Branched alkane index ($({A}_{1376})/{(A}_{1376}+{A}_{1460})$). During the upgrading process, molecules and heavy compounds of heavy crude oil are cracked, and it is predicted that after the upgrading process, more light compounds and less heavy compounds will be present. The results presented in Table 3 show that the upgrading process was carried out with the help of microwave waves in the presence of Fe_3_O_4_ nanocatalyst. According to the above results, the Aliphatic and Branched alkane index have increased and the Aromatic and Sulfoxide index has reduced. For an excellent evaluation of these modifications, the results are presented in Fig. 10.

**Table 3:** Functional group indexes of oil samples based on FTIR analysis result (*MW :Microwave).

	Crude oil	Crude oil + MW	Crude Oil + MW + Fe_3_O_4_ nanocatalysts synthesis by Co-precipitation	Crude Oil + MW + Fe_3_O_4_ nanocatalysts synthesis by MW
Aromatic index	0.295	0.286	0.275	0.256
Aliphatic index	0.563	0.655	0.672	0.683
Sulfoxide index	0.018	0.009	0.009	0.002
branched alkane index	0.188	0.201	0.202	0.214

As the results presented in Table 3, the indicators have changed during the process and the crude heavy oil has been upgraded. The aromatic and sulfoxide indices have decreased with microwave radiation, while the aliphatic and branched alkane indices have increased. On the other hand, the presence of Fe_3_O_4_ nanocatalyst has caused more synergy and upgrading in the samples. Due to Fig. 10, the Fe_3_O_4_ nanocatalyst synthesized by microwave-assisted compared to the Fe_3_O_4_ nanocatalyst synthesized using the co-precipitation method has a much more considerable effect on the desired indicators and has caused a more significant upgrade. So that the sample containing Fe_3_O_4_ nanocatalyst synthesized by microwave-assisted compared to the Fe_3_O_4_ nanocatalyst synthesized by the co-precipitation method has reduced the aromatic index by 13% and 7%, the sulfoxide index by 90% and 48%, as well as the aliphatic index by 21% and 19%, and the branched alkane index increased by 14% and 8%, respectively.

As a result of microwave radiation, due to the polarization or magnetization of Fe_3_O_4_ nanocatalysts and the orientation of electrons in the presence of electromagnetic waves, the molecular movements and oscillations of those particles will boost, and friction and heat will occur as a result of these movements and oscillations. The heat created through various transfer mechanisms is transferred to the oil-containing nanocatalysts and their surroundings. As the temperature increases, oil mobility increases and its viscosity decreases. In addition, the cracking and breaking of heavy molecules during the temperature increase into lighter components, and the upgrading of heavy oil occur. It should be noted that the catalytic effect of nanocatalysts on heavy molecules, their breakdown into lighter compounds, and the viscosity reduction and heavy oil upgrading.

## Conclusion

This research examined the synthesis of Fe_3_O_4_ nanocatalysts by microwave-assisted, the effect of parameters during synthesis, and the impact of nanocatalysts on heavy oil upgrading was investigated. The parameters of power and time of radiation of waves through the amount of heat produced are very effective and affect the synthesis process and the quality of the produced nanocatalysts. The presence of impurities in the crystallite network and the quality of the nanocatalyst were controlled by the power and time of wave radiation. As maintained by the XRD characterization, it could be concluded that with increasing irradiation time of more than 1 min and microwave radiation power of more than 400 watts, NH_4_Cl doped on the Fe_3_O_4_ nanocatalysts and reduce the purity of nanocatalysts. Because during the synthesis by microwave-assisted, the temperature increases with the growth in the power and radiation time of the waves, this increase in temperature causes doping NH_4_Cl on the Fe_3_O_4_ nanocatalysts. It is due mainly to the extensive ionic radius of NH_4_^+^ being larger than Fe_3_^+^.

Due to the electromagnetic properties of nanocatalysts, it was found that these nanocatalysts have the potential to absorb electromagnetic waves. Viscosity changes were investigated depending on microwave radiation (400 W) with 0.1% by weight and without Fe_3_O_4_ nanocatalysts and over time. By increasing the time of irradiation of waves, the viscosity of both samples decreased because the amount of heat produced during the process increased, leading to an increase in temperature, cracking of heavy components, and a decrease in the viscosity of the samples. On the other hand, the oil sample containing Fe_3_O_4_ nanocatalysts significantly decreases viscosity compared to the sample without nanocatalysts. Since Fe_3_O_4_ nanocatalysts are electromagnetic wave absorbers with a high ability to absorb electromagnetic waves and convert them into heat. The exhibition of heat and increase in temperature will crack the heavier compounds in the heavy oil and eventually reduce the samples' viscosity. It should be noted, since enough time has been given to the samples to cool down and stabilize the conditions, this decrease in viscosity will be stable.

In addition, the effect of the synthesis method (microwave-assisted method and co-precipitation method) on the efficiency and quality of nanocatalysts in the process of electromagnetic heating with microwave waves was investigated. In 4 min and 0.1% by weight of nanocatalysts, the effect of the nanocatalyst synthesis method and the presence of waves were investigated. Fe_3_O_4_ nanocatalysts synthesized with the microwave-assisted compared to nanocatalysts synthesized by the co-precipitation method have smaller sizes and caused a significant reduction in viscosity (by 28% and 23%, respectively); thus, these nanocatalysts at heavy oil upgrading are more effective for microwave heating and heavy oil upgrading. This difference could be because nanocatalysts synthesized with the microwave-assisted have a smaller crystallite size and more prominent absorbing microwaves than nanocatalysts synthesized by the co-precipitation method. The smaller crystallite size has increased the efficiency and the ability to absorb waves. On the other hand, by examining the results of FTIR analysis and functional index group, it can be seen that the presence of Fe_3_O_4_ nanocatalyst causes synergy and increases the efficiency of the microwave heating process, and in addition to reducing the viscosity of oil, it causes further heavy oil upgrading, reducing heavy compounds and increasing light compounds. Without the presence of Fe_3_O_4_ nanocatalyst and only with microwave radiation, the aromatic index and sulfoxide index decreased by 3% and 48%, respectively, and the alphabetic index and the branched alkane index increased by 16% and 7%, respectively. As it was presented, the synthesis method of Fe_3_O_4_ nanocatalyst has an essential effect on their efficiency, and this matter was confirmed according to the results of the FTIR analysis and the desired indicators. In the presence of microwave waves with a power of 400 watts and a duration of 4 min, the Fe_3_O_4_ nanocatalyst synthesized by microwave-asissted compared Fe_3_O_4_ nanocatalyst synthesized using co-precipitation has a more significant effect in reducing the aromatic index (13% vs. 7%) and the sulfoxide index (90% vs. 48%). Also, this condition was seen in the aliphatic and branched alkane indexes.

In the mentioned conditions and in the sample containing Fe_3_O_4_ nanocatalyst synthesized by microwave-assisted, the aliphatic index (21% vs. 19%) and the alkane branch index (14% vs.p 8%) increased more. Therefore, nanocatalysts synthesized microwave-assisted are more efficient and effective in the process of microwave heating, reducing viscosity and upgrading heavy oil.

### Supplementary Information

Supplementary Information.

## Data Availability

The authors declare that all data generated or analysed during this study are included in this published article [and its Supplementary Information files].

## Abbreviations

DI: Deionized distilled water
API: American petroleum Institute
EM: Electromagnetic waves
XRD: X-Ray diffraction
FESEM: Field emission scanning electron microscope
FTIR: Fourier transform infrared
