# Supplementary material for: Assessment of the synthesis method of Fe3O4 nanocatalysts and its effectiveness in viscosity reduction and heavy oil upgrading
Source: Sci Rep. 2023 Oct 24;13:18151. doi: 10.1038/s41598-023-41441-6 (PMC10598015; doi:10.1038/s41598-023-41441-6)
Supplement: Supplementary file 1 — Supplementary Information. [file 41598_2023_41441_MOESM1_ESM.docx]

1. **Determination of crystallite size by analysis**

The crystallite size of magnetite nanocatalyst would be estimated by the Scherer equation (eq.s1)

$$d=\frac{K\lambda}{\beta cos\theta} (S1)$$

Where d is the mean crystallite size of the magnetite nanocatalyst, K is the dimensionless shape coefficient, which is usually between 0.89-0.9; β is the half-maximum full width (FWHM). The peak is set at half the maximum intensity, λ X-ray diffraction wavelength. The average crystallite size of magnetite nanocatalyst synthesized using the Scherer equation in different synthesis conditions is estimated between 8 and 12 nm.

1. **Examination of the influence of irradiation time and power in microwave-assisted synthesis**

Since in the first stage of the synthesis, the synthesis by power conditions of 400 watts and the irradiation time of 1 minute had a satisfactory result and was free of impurities, the repeatability of this synthesis was investigated. On the other hand, considering that by raising the irradiation time of microwave waves during synthesis to more than 1 minute and the irradiance to more than 400 watts, to ensure the effect of power and irradiation time in microwave-assisted synthesis, two syntheses were performed with the conditions presented in Table 1-S

| No. | Power of radiation (watt) | Time of radiation (min) | Purity of Fe_3_O_4_ (%) |
| --- | --- | --- | --- |
| 1 | 400 | 1 | 100 |
| 2 | 600 | 1 | 89.1 |
| 3 | 800 | 0.5 | 94.6 |

Table 1-S-condition of synthesis, purity and average size in each of the samples

1. **Data analysis and ANOVA table**

As stated, the synthesis of magnetite nanocatalyst was carried out according to the general factorial design. The results of the ANOVA table are presented in Table 2-S. The F value indicates that the model is significant at less than 0.05%. It should be noted that the linear model is used (R-Squared: 0.869, Adj R-Squared: 0.7817). Figure 1-S illustrates the predicted versus actual values of purity of magnetite nanocatalyst. The scatter of points around the diagonal line indicates a sufficient correlation between the experimental data and the predicted values, confirming the model's robustness.

| **Source** | **Sum of squares** | **Df** | **Mean square** | **F-Value** | **p-value prob > F** | **Model Significance** |
| --- | --- | --- | --- | --- | --- | --- |
| **Model** | 266.70 | 2 | 133.35 | 9.95 | 0.0474 | significant |
| **A-time** | 153.16 | 1 | 153.16 | 11.43 | 0.0431 |  |
| **B-power** | 113.54 | 1 | 113.54 | 8.47 | 0.0619 |  |

Table 2-S- Analysis of variance (ANOVA) for selected general factorial model


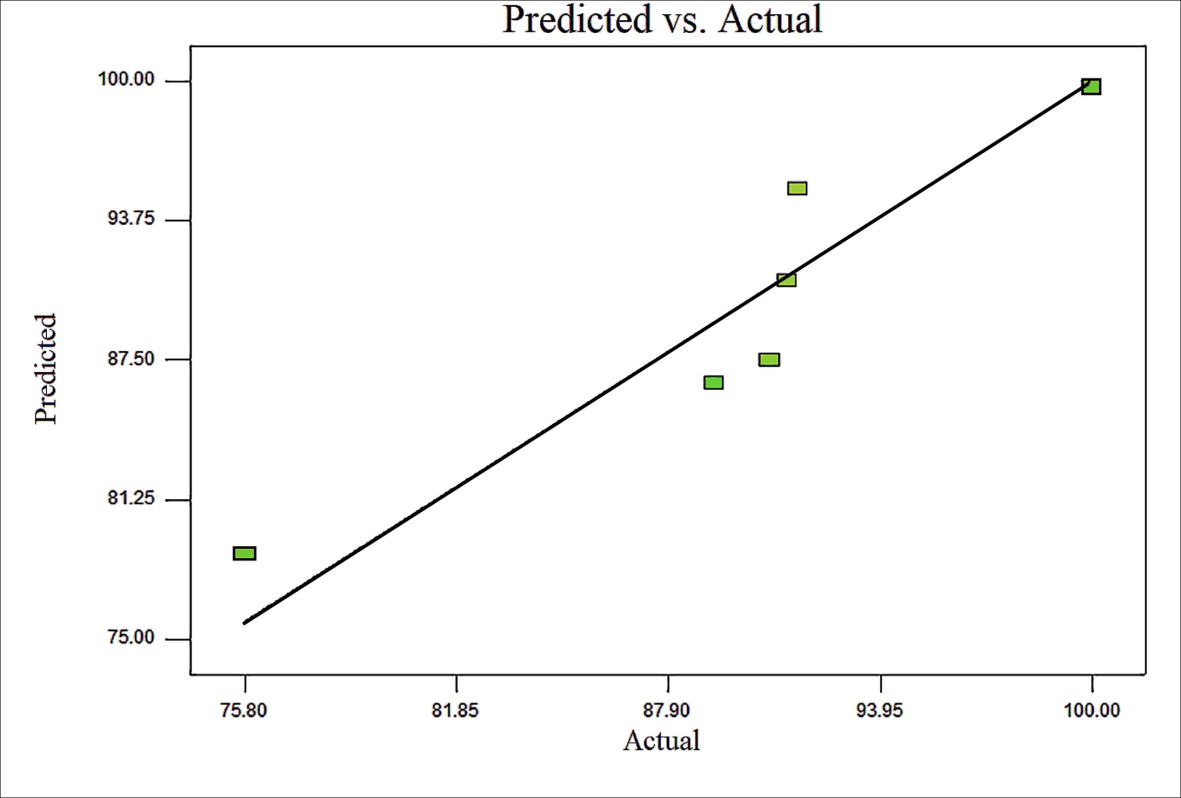


Figure 1-S- Predicted versus actual values of purity of magnetite nanocatalyst

**Data availability**

The authors declare that, all data generated or analyzed during this study are included in this published article [and its supplementary information files].
